# Toward better technology for social health in dementia: a review of implementation recommendations and research priorities to equitably meet the demand

**DOI:** 10.3389/fpubh.2026.1767647

**Published:** 2026-04-28

**Authors:** Dorota Szcześniak, Mauricio Molinari-Ulate, David Peter Neal, Sara Laureen Bartels, Angie Alejandra Diaz Baquero, Efrain David Rujano Vasques, Jochen René Thyrian, Manuel Franco-Martin, Karin Wolf-Ostermann, Joanna Rymaszewska, Louise Hopper, Rose-Marie Dröes

**Affiliations:** 1 Department of Psychiatry, Wroclaw Medical University, Wroclaw, Poland; 2 Escuela de Psicología, Universidad de Costa Rica, San José, Costa Rica; 3 Department of Medical Informatics, Amsterdam University Medical Centers, Amsterdam, Netherlands; 4 Department of Psychiatry and Neuropsychology and Alzheimer Centrum Limburg, Mental Health and Neuroscience Research Institute, Maastricht University, Maastricht, Netherlands; 5 Department of Personality, Assessment and Treatment, University of Salamanca, Salamanca, Spain; 6 Institute of Biomedical Research of Salamanca, University of Salamanca, Salamanca, Spain; 7 German Center for Neurodegenerative Diseases (DZNE), site Rostock/Greifswald, Greifswald, Germany; 8 Full Professor of Psychopathology and Mental Health in Salamanca University and Head of Psychiatry and Mental Health Department of Zamora Hospital, Zamora, Spain; 9 Institute of Public Health and Nursing Research (IPP) and High-Profile Area of Health Sciences, University of Bremen, Bremen, Germany; 10 Department of Clinical Neurosciences, Faculty of Medicine, Wroclaw University of Science and Technology, Wroclaw, Poland; 11 School of Psychology, Dublin City University, Dublin, Ireland; 12 Department of Psychiatry, Amsterdam University Medical Centers, Amsterdam, Netherlands

**Keywords:** best practice guidance, dementia, effectiveness, implementation, personalisable technology, research recommendations, usability

## Abstract

**Background:**

Social health is increasingly recognized as a key dimension of wellbeing in dementia, yet it remains unclear to what extent available assistive technologies are personalisable, usable, and effective in addressing related unmet needs.

**Objective:**

This umbrella review aimed to (1) identify how many and which technologies are personalisable, usable and effective in supporting social health in dementia, and (2) synthesize recommendations from the current literature on how to improve equitable implementation.

**Methods:**

An explorative review of reviews was conducted, including 28 reviews published between 2007 and 2024. The INDUCT/DISTINCT Best Practice Guidance for Human Interaction with Technology in Dementia was also included. Technologies and recommendations were analyzed using a combined frequency-based and thematic approach and categorized according to the three social health domains (fulfilling potential and obligations; managing life with some degree of independence; participation in social activities) and across micro-, meso-, and macrolevels.

**Results:**

Of all technologies discussed, 48% were reported as personalisable and 59% as usable. However, only 23% of personalisable technologies demonstrated effectiveness in at least one randomized controlled trial. Most evidence related to the domain of managing life with some degree of independence, while fewer technologies showed demonstrated effectiveness for fulfilling potential or enhancing social participation. Recommendations primarily addressed implementation strategies, equity considerations, and stakeholder collaboration. Future research priorities included the development of needs-based, personalisable, and diversity-sensitive technologies, improved methodological rigor, and supportive policy and funding structures.

**Conclusions:**

While half of the technologies are described as adaptable to user needs, preferences or abilities and more than half as usable, robust evidence for their effectiveness in promoting social health remains limited. Clearer operationalisation of personalisation, stronger evaluation designs and improved implementation strategies are needed to ensure that people with dementia can equitably access technologies that promote their social health.

## 1 Introduction

In the care for people with dementia, there is an increasing emphasis on the importance of social health (1). In this review, social health is understood as the capacity of individuals to *fulfill their potential and obligations, manage their lives with some degree of independence, and participate in meaningful social activities* (2). The concept of social health goes beyond cognitive and medical conditions and focuses on the individual's ability to maintain wellbeing through social participation and adaptive engagement (1, 3). Indeed, there is a broader transformation in the field of care for people with chronic diseases—from a narrow focus on disease-related deficits to a more holistic perspective that recognizes the interplay between individual capabilities to adjust and the social environment in shaping quality of life (4). Yet, dementia poses profound challenges to social health: people living with dementia and their family caregivers face increased risks of social isolation, reduced social participation, and communication difficulties, which lead to feelings of loneliness (5, 6). In addition to social withdrawal, people with dementia also experience behavioral and mood complaints, such as apathy, aggressiveness, disinhibition, irritability, anxiety, depression and emotional lability, and thinking disorders like paranoid ideas of being at risk of assault or losing all belongings and more. These complaints may be associated with brain degeneration or the way people cope with its consequences, and/or the interaction with their social environment. They can further limit social engagement (7).

As populations age globally, the number of people with dementia is expected to double in the coming three decades. There is therefore a growing and urgent demand for effective, scalable solutions to support *social health* in dementia. A wide array of technological interventions has emerged, including information and communication technologies (ICT), digital platforms, artificial intelligence (AI), and advanced communication networks, designed to promote social participation, facilitate connection, and enhance quality of life for people with dementia and their caregivers (8–12). In this review, we define personalisable technologies as assistive or digital tools that can be adapted to the individual needs, preferences, and abilities of people with dementia and their caregivers. Such adaptation may involve adjustable interfaces, tailored content, or flexible modes of interaction. Personalisability therefore refers to responsiveness to individual differences rather than a uniform, one-size-fits-all design. An expert-based consensus published by the Dutch knowledge organization for care and support Vilans (13), provides a helpful theoretical framework that categorizes the wide range of technologies with regard to specific uses, and users (e.g. people with dementia or (in)formal caregivers) across the dementia journey. Technologies aimed at social health may help improve mental health, i.e. decreasing behavioral and mood symptoms, by facilitating meaningful and satisfying interaction and emotional stimulation (14, 15). Exploratory studies evaluating such technologies have highlighted their potential to foster communication and interaction with people with dementia (14–16). Many technology users report entertainment value and ease of use, factors that together enhance acceptance and create positive user experiences (16). While qualitative research points to the potential of technology to reduce loneliness, enhance wellbeing, and promote social interaction, quantitative *evidence on effectiveness* remains sparse and frequently inconclusive (10, 11, 17, 18, 91, 92). Dementia-specific reviews have similarly highlighted the limited and inconclusive evidence regarding the impact of technological interventions on social participation and social isolation outcomes; studies are often limited in time and scale, rely on heterogeneous methodologies, and lack standardized outcome measures, making it difficult to draw generalizable conclusions (8, 18, 19). Previous reviews have also suggested that more robust methodological designs should be applied (8, 18).

Despite their promise, technologies in dementia care also face significant *implementation challenges*. Barriers such as limited digital literacy, unequal access to technological infrastructure, and the absence of sufficiently tailored solutions often prevent meaningful adoption by people living with dementia and their support networks (9, 20, 21). The use of technology can also change care procedures, causing resistance from caregivers. In response to these challenges, some studies have explored technological adaptations aimed at improving the *accessibility and usability* of technology for this population. These efforts include strategies to enhance communication interfaces, such as the application of conversational techniques to support interactions with social robots (22), as well as broader approaches like software optimization, emotion modeling (e.g., sadness, anger, happiness), and social training programs (23).

Although some technologies are described as adaptable or customisable, the degree to which these tools are truly *personalisable*–in the sense that technology configurations or interfaces are responsive to individual needs, preferences, and lived experiences-remains unclear. Literature reviews have emphasized the importance of tailoring technologies to the specific needs of the dementia population (24, 25). Personalization is particularly crucial for supporting individuals' ability to adapt to new technologies, which can be challenging in the context of cognitive decline (26). Design choices that overlook this need may negatively impact usability and user experience-both of which are essential for ensuring that people with dementia can effectively engage with technologies (27).

In addition, other implementation issues related to equity must be considered. Innovations are often piloted in resource-rich environments but fail to scale sustainably or reach underserved populations. Without addressing these structural and social barriers, technological interventions may deepen existing disparities rather than mitigate them (28). These limitations underscore the need for more rigorous evaluations and a clearer understanding of how, and under what conditions, technological interventions can equitably and effectively support social health in dementia.

The review of reviews we report on in this paper was conducted by the Technology Subgroup of the INTEREST Working Group (INnovaTions in divERsity and Equity in Social health research in DemenTia), funded by the European Joint Programme Neurodegenerative Diseases Research (JPND) (29). The aim was to get insight in what needs to be done, and further investigated, to ensure that the supply of usable and effective technology to promote social health in dementia equitably meets the demand. We focused on assistive technologies (AT) which can support people with dementia and their family caregivers in a tailored way with respect to their self-identified unmet needs (including memory support, social contact/company, daytime meaningful activities, safety, psychological distress, and information about their condition), where the present supply of health and social care does not fulfill the demand (19, 30–32).

Research questions were:

(1) How many, and which, technologies are personalisable and effective in one or more of the most frequently mentioned unmet need areas related to social health?
(2) What needs to be done and further investigated, according to the current literature, to ensure that the supply of usable and effective technology to promote physical, mental, and social health in dementia equitably meets the demand?

This review of reviews builds on research conducted within existing European networks, namely the INTERDEM Assistive Technology Taskforce (8, 33), and the European Marie Sklodowska-Curie funded INDUCT and DISTINCT Innovative Training Networks (2), in which 30 PhD students in eight countries participated, as well as more than 60 experts in the field. INDUCT and DISTINCT specifically addressed in the context of dementia, the development, evaluation and implementation of assistive technology in everyday life, technology for meaningful activities, healthcare technology, and technology specifically for the three social health domains. These two programmes therefore represent a concentrated body of knowledge and expertise directly relevant to the aims of this review.

## 2 Methods

### 2.1 Design and included reviews and other sources

An explorative, review of reviews into effective and personalisable technology that aim to promote social health in dementia was conducted (umbrella review). *Inclusion criteria* were that the review was i) at least partly focused on people with dementia and ii) aspects of their social health, and that iii) specific AT were mentioned. *Exclusion criteria* were that iv) the review did *not* focus on dementia, v) that it was targeted on informal or professional caregivers only, or vi) that (almost) no specific technologies were mentioned. In total, 28 reviews on the application and evaluation of technology in dementia care were included for analyses (see 3. Results). Eight of these reviews were published by researchers of the INDUCT and DISTINCT Networks which focused on the development, evaluation and implementation, of assistive technologies in dementia. In addition to the reviews conducted within INDUCT and DISTINCT, another 20 reviews which are referred to in the literature from 2007 until 2024 were reviewed. Most of these were identified in the systematic reviews conducted by the INTERDEM Technology Taskforce in 2017 (8) and in 2024 (33) and through literature searches conducted by the INTEREST Needs Subgroup.

To answer the second research question, the INDUCT/DISTINCT Best Practice Guidance for Human Interaction with Technology in Dementia [(2); www.dementiainduct.eu/guidance/], containing the recommendations from the research done by the INDUCT and DISTINCT networks, was also searched for relevant recommendations related to the research focus (tailored support, personalisable, usable and effective technology, implementation on a micro, meso, and macrolevel, equity).

### 2.2 Data extraction

The following data were extracted from included reviews: year of publication, type of study, specific technologies named, and for each named technology, the mentioned target group(s), unmet needs, social health domain, personalisability/possibility of tailored application, tested usability, effectiveness on social health, mental health, or quality of life, and recommendations for implementation and further research on a micro-(user), meso-(organizational), and macrolevel (policy, laws and regulations).

We also extracted recommendations on implementation and directions for future research related to the topics mentioned above, with special attention for (prevention of) inequity. Recommendations were mapped to the three social health domains on a micro-, meso- and macrolevel. The following definitions for the Social health domains were used (2):

(i) Capacity to fulfill one's potential and obligations: the ability of a person (living with or caring for a person with dementia) to function in the society according to their competencies and talents (“potentials”) in the best possible way and to meet social demands (“obligations”) on a micro and macro societal level;
(ii) Ability to manage life with some degree of independence: the ability to preserve autonomy and to solve problems in daily life, as well as to adapt to and cope with the practical and emotional consequences of dementia;
(iii) Participation in social activities: the act of being occupied or involved with meaningful activities and social interactions and having social ties and relationships, which are meaningful to the person living with dementia themselves (2).

### 2.3 Procedure

Included reviews were reviewed by pairs of reviewers: a first reviewer performed data extraction and extracted data were checked by a second reviewer. In case of missing data or disagreement between reviewers, data were added or discussed respectively until consensus was reached. In total, there were eight reviewers (ADB, DN, DS, SLB, JR, MMU, KWO, RMD, SB), all dementia care researchers with expertise in social health and technology.

The recommendations from the INDUCT/DISTINCT Best Practice Guidance were extracted by four reviewers (RMD, SLB, JR, RT), also in pairs of first and second reviewer, where the first reviewer performed data extraction and categorized the recommendations into the social health domains and micro-, meso-, and macrolevel, and the second reviewer checked the categorization. In case of disagreement between reviewers, data were added or discussed respectively until consensus was reached.

### 2.4 Analyses

To address the research questions, we used a combined frequency-based content analysis and thematic analysis approach. The following analyses were undertaken:

How many, and which, technologies are personalisable and effective in one or more of the most frequently mentioned unmet need areas? (research question 1)

Descriptive statistics were used, specifically: number and percentage of personalisable AT of all discussed AT per review; mean percentage of all reviews; and the number and percentage of personalisable AT in Social Health domain 1, 2, and 3 (overall, for all reviews together). Features of AT that facilitated personalization (for all technologies of all SH domains together) were narratively synthesized. Furthermore, we calculated the number and percentage of personalisable AT with respect to which usability was mentioned to have been tested in the target group, and the number and percentage of personalisable AT with demonstrated effectiveness in controlled trials. Finally, we calculated the number and percentage of demonstrated effective, personalisable AT divided into the Social Health Domains 1, 2, 3.

What needs to be done and further investigated, according to the current literature, to ensure that the supply of usable and effective technology to promote physical, mental and social health in dementia equitably meets the demand? (research question 2)

Recommendations regarding *personalization and implementation* of AT made in the INDUCT/DISTINCT Best Practice Guidance (87) and the selected reviews were also categorized per social health domain, and on a micro-, meso-, and macrolevel, following the framework proposed by Dröes et al. (2). This framework was used because it allows findings to be structured across different layers of implementation, relevant to different stakeholder groups (e.g., people with dementia and (in)formal caregivers, organizations, and policy-level actors). Recommendations for *future research* into AT made in the INDUCT/DISTINCT Best Practice Guidance and the included reviews, as well as the review of reviews of Meiland et al. (8), were thematically analyzed. Within these themes it was indicated whether recommendations concerned the micro, meso or macrolevel.

## 3 Results

### 3.1 Review characteristics

**Figure 1:**
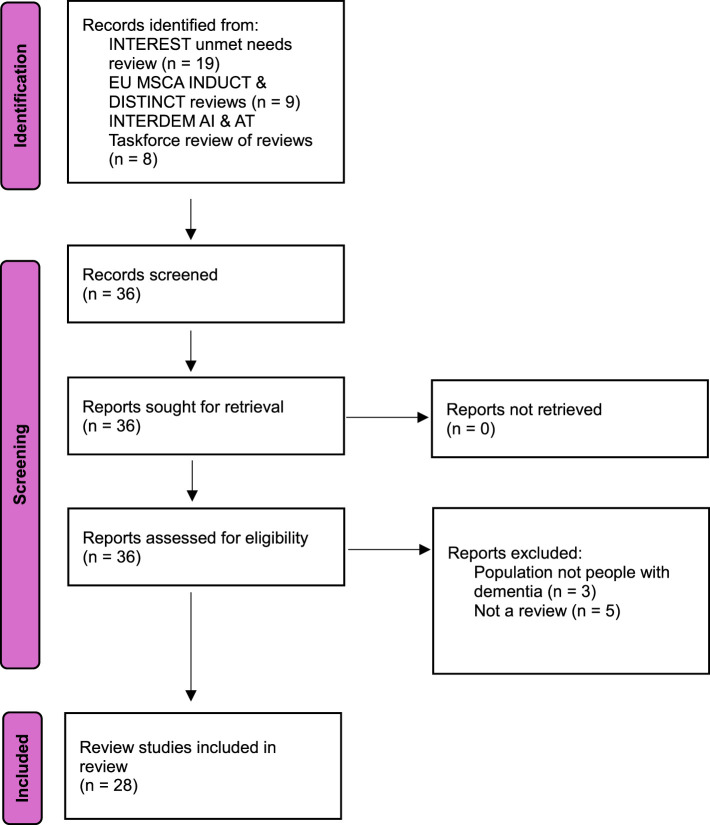
Flowchart overview of record identification, screening and included review studies.

In total, 36 reviews concerning AT were identified from the sources described in the methods for screening, as shown in Figure 1.

Of these, 28 reviews were included for further analysis (Table 1). The excluded eight reviews either concerned different target populations, such as professional caregivers or informal caregivers only (*n* = 3), or were not suitable for extracting data on specific technologies because they were not reviews, or were reviews of reviews (*n* = 5). Whilst excluded from the review and not considered in addressing research question 1, to answer research question 2, we also included the INTERDEM position paper of Meiland et al. (8), because it contained relevant recommendations on implementation of technology in practice and directions for future research.

**Table 1:** Characteristics of included reviews.

Study	Nature of review (rapid, scoping, mapping, systematic)	Eligible designs of primary studies (qualitative, quantitative, mixed methods, reviews)	Method of synthesis (narrative, meta-analysis)	Number of primary studies included
Barbosa et al., 2024 (90)	Rapid review	Qualitative, quantitative, mixed methods and reviews	Narrative	18
Brims and Oliver, 2019 (38)	Systematic review	Quantitative, mixed methods	Meta-analysis	3
Buele et al., 2023 (39)	Rapid review^*^	Qualitative, quantitative, mixed methods	Narrative	8
Caprioli et al., 2023 (89)	Systematic review	Qualitative, quantitative, mixed methods and reviews	Narrative	17
Chae et al., 2023 (86)	Systematic review	Quantitative, mixed methods	Meta-analysis	44
Cho et al., 2023 (88)	Systematic review	Quantitative, mixed methods	Meta-analysis	16
Hardy et al., 2024 (40)	Scoping review	Qualitative, quantitative, mixed methods	Narrative	39
Heins et al., 2021 (17)	Systematic review	Qualitative, quantitative, mixed methods	Narrative	36
Henk-Verloo et al., 2021 (41)	Scoping review	Qualitative, quantitative, mixed methods	Narrative	12
Hill et al., 2024 (42)	Mapping review	Qualitative, quantitative, mixed methods and reviews	Narrative	48
Hirt et al., 2019 (43)	Scoping	Qualitative, quantitative, mixed methods and reviews	Narrative	24
Hoel et al., 2021 (10)	Systematic review	Qualitative, quantitative, mixed methods	Narrative	18
Kim et al., 2021 (44)	Scoping review	Qualitative, quantitative, mixed methods and reviews	Narrative	12
Klimova et al., 2016 (45)	Rapid review^*^	Qualitative, quantitative, mixed methods and reviews	Narrative	33
Koo et al., 2019 (46)	Scoping review	Qualitative, quantitative, mixed methods and reviews	Narrative	24
Lauriks et al., 2007 (47)	Rapid review^*^	Qualitative, quantitative, mixed methods	Narrative	46
Lee et al., 2021 (48)	Systematic review	Qualitative, quantitative, mixed methods	Narrative	11
Monnet et al., 2024 (49)	Systematic review	Qualitative, quantitative, mixed methods	Narrative	7
Neal et al., 2021 (11)	Systematic review	Quantitative, mixed methods	Narrative	9
Oksnebjerg et al., 2020 (50)	Scoping review	Qualitative, quantitative, mixed methods	Narrative	8
Papaioannou et al., 2022 (51)	Systematic review	Quantitative, mixed methods	Meta-analysis	20
Saragih et al., 2021 (52)	Systematic review	Quantitative, mixed methods	Meta-analysis	12
Swinnen et al., 2022 (53)	Systematic review	Quantitative, mixed methods	Narrative	8
Tan et al., 2023 (54)	Systematic review	Quantitative, mixed methods	Narrative	8
Topo et al., 2009 (55)	Rapid review^*^	Qualitative, quantitative, mixed methods	Narrative	46
van Santen et al., 2018 (56)	Systematic review	Quantitative, mixed methods	Narrative	3
Vermeer et al., 2019 (57)	Scoping review	Qualitative, quantitative, mixed methods	Narrative	28
Yi et al., 2021 (58)	Systematic review	Qualitative, quantitative, mixed methods	Narrative	17

^*^Authors of reviews marked did not specify the nature of the review, classification as a rapid review was based on our assessment of the methods.

### 3.2 Identified personalisable, effective technologies for social health in dementia

How many, and which, technologies are personalisable and effective in one or more of the most frequently mentioned unmet need areas? [Research question 1]

Table 2 shows for each included review, how many specific technologies were discussed, how many of these were personalisable, for how many of the personalisable technologies there was evidence of usability, and for how many there was evidence from at least one randomized controlled trial of effectiveness.

**Table 2:** Number of mentions of specific technologies from included reviews that were considered personalisable, usable, and effective.

Review	Mentioned technologies *n*	Mentioned personalisable technologies *n* %		Personalisable technologies, usability studied *n* %		Personalisable technologies, effective in RCT *n* %	
1. Barbosa et al., 2024 (90)	10	1	(10)	0	(0)	0	(0)
2. Brims and Oliver, 2019 (38)	3	0	(0)	0	(0)	0	(0)
3. Buele et al., 2023 (39)	1	0	(0)	0	(0)	0	(0)
4. Caprioli et al., 2023 (89)	17	12	(71)	9	(75)	1	(8)
5. Chae et al., 2023 (86)	25	10	(40)	5	(50)	8	(80)
6. Cho et al., 2023 (88)	16	9	(56)	4	(44)	6	(66)
7. Hardy et al., 2024 (40)	40	28	(70)	5	(18)	3	(11)
8. Heins et al., 2021 (17)	3	1	(33)	0	(0)	1	(100)
9. Henk-Verloo et al., 2021 (41)	11	6	(55)	6	(100)	0	(0)
10. Hill et al., 2024 (42)	12	2	(17)	2	(100)	1	(50)
11. Hirt et al., 2019 (43)	6	4	(67)	4	(100)	0	(0)
12. Hoel et al., 2021 (10)	17	10	(59)	6	(60)	1	(10)
13. Kim et al., 2021 (44)	12	5	(42)	0	(0)	0	(0)
14. Klimova et al., 2016 (45)	3	0	(0)	0	(0)	0	(0)
15. Koo et al., 2019 (46)	15	7	(47)	6	(86)	0	(0)
16. Lauriks et al., 2007 (47)	40	9	(23)	7	(78)	1	(11)
17. Lee et al., 2021 (48)	8	8	(100)	6	(75)	0	(0)
18. Monnet et al., 2024 (49)	3	3	(100)	3	(100)	0	(0)
19. Neal et al., 2021 (11)	8	5	(63)	3	(60)	3	(60)
20. Oksnebjerg et al., 2020 (50)	8	7	(88)	5	(71)	0	(14)
21. Papaioannou et al., 2022 (51)	4	3	(75)	1	(33)	2	(67)
22. Saragih et al., 2021 (52)	12	0	(0)	0	(0)	0	(0)
23. Swinnen et al., 2022 (53)	8	8	(100)	6	(75)	6	(75)
24. Tan et al., 2023 (54)	4	1	(25)	0	(0)	0	(0)
25. Topo et al., 2009 (55)	20	4	(20)	1	(25)	1	(25)
26. van Santen et al., 2018 (56)	3	2	(67)	1	(50)	2	(100)
27. Vermeer et al., 2019 (57)	8	1	(13)	1	(100)	0	(0)
28. Yi et al., 2021 (58)	12	12	(100)	12	(100)	0	(0)
**Total**	329	158	(48)	93	59	36	23

#### 3.2.1 Personalization aspects

In total, 329 mentions of specific technologies were identified from the included reviews (range 1–40, per review), of which 158 mentions (48%) were of technologies considered to be to some extent personalisable (range 0%−100%, per review). The most common ways in which technologies were considered to offer some degree of personalization were in *adjustable settings*, for example, in how screen-based applications were displayed, or in *flexible content*, for example, the ability to add or remove specific apps or features to a software-based technology. Personalization was reported to be necessary, and in some cases actually provided, in response to variations on factors related to the *person and their life history* (to provide content from the individual's own life; to provide culturally appropriate content based on age, interests or religion; to adapt technology to their level of digital literacy), and to their *physical and mental health status* (severity of cognitive impairment; presence of dementia-related symptoms such as apathy or agitation; their physical and aerobic fitness).

#### 3.2.2 Personalisable technologies per Social health domain

In relation to the three domains of social health, Table 3 shows that the number of mentions of specific technologies that could support capacity to fulfill one's potential and obligations was 83, of which 48 (58%) were considered personalisable. Managing life with some degree of independence was supported by 193 mentions of technologies, of which 97 (50%) were considered personalisable. There were 130 mentions of technologies that could support participation in social activities, of which 62 (48%) were considered personalisable.

**Table 3:** Number of mentioned technologies, and number of which were considered personalisable, and considered relevant to each domain of social health.

	Social health domain 1			Social health domain 2			Social health domain 3		
**Review**	**Mentioned technologies** ***n***	**Personalisable technologies** ***n*** **%**		**Mentioned technologies** ***n***	**Personalisable technologies** ***n*** **%**		**Mentioned technologies** ***n***	**Personalisable technologies** ***n*** **%**	
1. Barbosa et al., 2024 (90)	1	0	(0)	5	1	(20)	10	1	(10)
2. Brims and Oliver, 2019 (38)	0	0	(0)	3	0	(0)	0	0	(0)
3. Buele et al., 2023 (39)	0	0	(0)	1	0	(0)	0	0	(0)
4. Caprioli et al., 2023 (89)	8	5	(63)	16	11	(69)	3	1	(33)
5. Chae et al., 2023 (86)	19	9	(47)	9	4	(44)	4	3	(75)
6. Cho et al., 2023 (88)	0	0	(0)	3	3	(100)	14	7	(50)
7. Hardy et al., 2024 (40)	11	7	(64)	5	2	(40)	25	19	(76)
8. Heins et al., 2021 (17)	0	0	(0)	2	0	(0)	3	1	(33)
9. Henk-Verloo et al., 2021 (41)	3	2	(67)	8	4	(50)	3	2	(67)
10. Hill et al., 2024 (42)	1	1	(100)	11	2	(18)	1	0	(0)
11. Hirt et al., 2019 (43)	3	2	(67)	6	4	(67)	3	3	(100)
12. Hoel et al., 2021 (10)	0	0	(0)	0	0	(0)	17	10	(59)
13. Kim et al., 2021 (44)	0	0	(0)	5	5	(100)	0	0	(0)
14. Klimova et al., 2016 (45)	0	0	(0)	3	0	(0)	0	0	(0)
15. Koo et al., 2019 (46)	8	4	(0)	12	5	(42)	4	2	(0)
16. Lauriks et al., 2007 (47)	14	6	(43)	31	9	(29)	14	2	(14)
17. Lee et al., 2021 (48)	1	1	(100)	6	6	(100)	3	3	(100)
18. Monnet et al., 2024 (49)	0	0	(0)	3	3	(100)	3	3	(100)
19. Neal et al., 2021 (11)	0	0	(0)	8	5	(63)	1	1	(100)
20. Oksnebjerg et al., 2020 (50)	0	0	(0)	8	7	(88)	2	2	(100)
21. Papaioannou et al., 2022 (51)	0	0	(0)	4	3	(75)	0	0	(0)
22. Saragih et al., 2021 (52)	2	0	(0)	2	0	(0)	9	0	(0)
23. Swinnen et al., 2022 (53)	0	0	(0)	5	5	(100)	0	0	(0)
24. Tan et al., 2023 (54)	0	0	(0)	0	0	(0)	3	1	(33)
25. Topo et al., 2009 (55)	1	0	(0)	14	3	(21)	8	1	(13)
26. Van Santen et al., 2018 (56)	0	0	(0)	3	2	(67)	0	0	(0)
27. Vermeer et al., 2019 (57)	0	0	(0)	8	1	(13)	0	0	(0)
28. Yi et al., 2021 (58)	11	11	(100)	12	12	(100)	0	0	(0)
**Total**	83	48	58	193	97	50	130	62	48

#### 3.2.3 Usability of personalisable technologies

Of the 158 mentions of technologies that were personalisable, information about usability of the technology was found associated with 93 (59%), though this was generally reported with only very limited details in the reviews and original articles regarding methods and findings (Tables 2, 4).

**Table 4:** Summary of RCT results in relation to personalisable technologies referenced in included reviews.

Referenced RCT result (*n* = 27)	Technology	Relevant social health domain(s)	Sample size	Outcome(s) vs. control	Evidence of usability
Bamidis et al., 2014 (85)	FitForAll exergames	2	322	Improved cognition across several domains	Feasibility asserted
Camberg et al., 1999 (59)	Audio and videotapes	3	54	Decreased agitation and withdrawal	All subjects accepted the intervention
Cavallo and Angilletta, 2019 (60)	Individual computerized cognitive training	1	80	Reduced neuropsychological symptoms	None reported
D'Aniello et al., 2021 (61)	Web-based music app	3	60	Reduced neuropsychological symptoms	None reported
Davison et al., 2016 (62)	Memory box touchscreen PC	3	11	Reduced depression and anxiety	Memory Box was well utilized and highly rated by residents, families and staff members.
Dodge et al., 2015 (63)	Video-call via PC	1, 3	83	Improved psychomotor speed	None reported
Galante et al., 2007 (64)	Neuropsychological training by Tonetta	1	11	Improved performance on cognitive exercises	None reported
Garland et al., 2007 (65)	Video and audio tapes	1	30	Reduced physical and verbal expressions of agitation	None reported
Karssemeijer et al., 2019 (66)	Bike labyrinth cognitive aerobic exergame	2	115	Improved psychomotor speed	Feasibility asserted, higher adherence reported than in control group.
Lazarou et al., 2019 (67)	Intelligent home monitoring system combined with tailored non-pharmacological interventions	1, 2	18	Improved cognitive function, sleep quality and ADL scores	Feasibility asserted based on participant feedback
Lee et al., 2013 (68)	Computerized Errorless Learning based memory training	1	19	Improved cognition across several domains	None reported
Liao et al., 2020 (69)	Microsoft Kinect VR for Tai Chi and aerobic exercise	2	42	Improvement in self-management	None reported
Manav and Simsek 2019 (70)	YouTube videos	3	32	Improved cognitive function, reduced apathy	None reported
Manenti et al., 2020 (71)	Virtual reality rehabilitation system	1	49	Improved memory, language, visuo-constructional abilities	Overall, high rates of participant agreement, recruitment and treatment adherence supported the feasibility of both face-to-face and telerehabilitation interventions. Moreover, the analyses on system usability evidenced good usability of clinic-VRRS and Tele@H-VRRS
Moon and Park 2020 (72)	Tablet-based reminiscence therapy intervention	2, 3	49	Reduced depression and increased engagement	Usability tests indicated that the resolution of photos or videos, size of screen, and auto-play interval were acceptable. The appropriate length of the program and the volume of the sound were revised.
Nishiura et al., 2019 (73)	Electric calendar app for Android tablet	1, 2	27	Improved self-management, improved mini mental state examination score	Post-intervention interviews revealed that most participants had positive impressions of using the electric calendar. Most healthy older adults mentioned that electric calendars were useful.
Nousia et al., 2018 (74)	RehaCom software	1	50	Improvements in several cognitive domains	Suggested based on lack of dropouts
O'Sullivan et al., 2022 (75)	Tablet-based intervention	2, 3	162	Improved quality of life, reduced PRN psychotropic medication use	None reported
Padala et al., 2017 (76)	Exercise programme based on Wii-Fit interactive video game	2	30	Improved balance and reduced fear of falling	Ease of use asserted
Possin et al., 2019 (77)	Telephone and email support	1, 2	780	Increased PLWD's quality of life, reduced emergency department visits, reduced unpaid carers' depression and caregiving burden	97% reported that they would recommend the care ecosystem to another unpaid carers.
Sautter et al., 2021 (78)	It's never too Late, interactive touch screen	2, 3	28	Improved wellbeing, reduced systolic blood pressure	Feasibility asserted
Swinnen et al., 2021 (79)	Dividat Senso (exergaming)	2	55	Improved gait speed, mobility, balance, step reaction time, and cognitive function. Reduced depressive symptoms	None reported
Vanoh et al., 2019 (80)	WESIHAT 2.0 web-based wellness application	2	60	Improved informational support	Difficulties were reported in dealing with unknown people. The use of the computer required complex motor functioning, language processing, and focus.
Wiloth et al., 2017 (81)	Physiomat motor-cognitive exergame	2	99	Improved duration and accuracy in training	Asserted simplicity of design
Wilson et al., 2001 (82)	NeuroPage prosthetic memory and activity cueing	1, 2	143	Reduced memory and planning failures, improved ADL scores	None reported
Wittelsberger et al., 2013 (83)	Nintendo Wii Bowling	2	27	Improved upper body strength	Usability asserted
Yu et al., 2019 (84)	Memory matters mobile app for reminiscence therapy	3	80	Improved social interaction and reduced apathy	None reported

#### 3.2.4 Effectiveness of personalisable technologies

There were 36 mentions of personalisable products (23%) associated with evidence from at least one randomized controlled trial (RCT) for the effectiveness of the technology. Table 4 shows technologies with evidence from RCTs (*n* = 27) that were cited in at least one review included in this study (some were mentioned in more than one review), and a brief summary of the results of each study. Overall, nine RCTs of personalisable technologies showed no effect, implicating that of the in total 36 conducted RCTs 75% showed evidence for effectiveness.

There were 11 technologies with positive RCT results relevant to supporting capacity to fulfill potential and obligations (e.g. improved cognitive performance, psychomotor speed, and decreased neuropsychological symptoms), 15 technologies with positive RCT results relevant to support managing life with some degree of independence (e.g. improved self-management, cognition, activities of daily living, balance and reduced fear of falling, improved quality of life and wellbeing, reduced depression, psychotropic medication, and caregiver burden) and nine technologies with positive RCT results relevant to supporting participation in social and other meaningful activities (e.g. improved social interaction, decreased agitation and withdrawal, reduced neuropsychiatric symptoms like depression, anxiety, apathy, and reduced systolic blood pressure). There were eight technologies that were relevant to more than one social health domain.

### 3.3 Recommendations to match supply and demands equitably

What needs to be done and further investigated to ensure that the supply of usable and effective technology to promote physical, mental and social health in dementia equitably meets the demand? (Research question 2)

a) Recommendations on personalized implementation of technology for Social health in Dementia from the INDUCT/DISTINCT Best Practice Guidance for Human Interaction with Technology in Dementia ((2); ref BPG), from the included 28 reviews (ref 1–28, Table 2) and the Interdem position paper ((8); ref 0).

#### 3.3.1 General recommendations

On a *microlevel*, it is recommended that people with dementia and family caregivers should be provided clear and early information on technology (0, 11). Barriers to adoption and implementation should be considered when delivering web- and app-based interventions digitally to maximize the potential reach and effect on people living with dementia and their families (17). Psychosocial support for caregivers should be included in technology-based interventions (11). On a *mesolevel*, it is suggested that to inform their clients well, healthcare professionals should be (made) aware of current and new ICT developments and services (16) and that technology should be integrated into the built environment and routine healthcare practice (0), for example, integrate reminder services and assistance from caregivers (15). Adequate infrastructure for technology deployment should be ensured (0) and clinical evaluation of the usefulness, acceptability, and effectiveness of technological devices should be done (12). On a *macrolevel*, it is recommended to identify applications of particular relevance for people with dementia (0); clinicians, service providers, and policymakers, should seize the opportunities to support the technological transformation in dementia care (1), and policymakers should establish national standards for telehealth equipment to ensure quality and interoperability across platforms, and create guidelines that recognize and support the use of low-cost communication technologies.

#### 3.3.2 Personalisation

On a *microlevel*, it is recommended to meet individual variation in personal factors, like needs and abilities (0) and contextual factors (17), particularly to those that live alone or do not have supporters (1) or lack confidence in using technology (17), and address emotional states of users (0); to adopt the most effective technology-based intervention for behavior and mood symptoms in a personalized manner and develop an intervention protocol tailored to the individual and relevant target outcomes in the specific context (6). The characteristics of individuals who may benefit from robots, like PARO, need to be specified to enhance personalized and effective ways to improve wellbeing of people with dementia (6). On a *mesolevel*, AT producers are recommended to make user interfaces simpler and tailored to specific requirements of the (individual) person with dementia (11).

#### 3.3.3 Social health domain 1: capacity to fulfill one's potential and obligations

On a *microlevel*, it is recommended that for the use of “everyday”, “surveillance” and “cognitive training technology”, individual needs are considered and assistance provided, based on comprehensive assessment by, for example, an occupational therapist (BPG). Discrimination should be prevented by ensuring that technological systems (e.g. in parking locations) are usable for customers living with dementia (BPG). Web-based tools for decision making should use concrete scenarios to illustrate the information provided and consider length and amount of information (18). Additional support should be provided to facilitate the use of web-based tools for people with dementia and their caregivers (18). Virtual Reality (VR) activities should be tailored to individual needs and capabilities and physical condition to avoid injury or loss of motivation, necessitating adjustments in task difficulties, session duration, and supervision during training (most study-participants have been older adults with primary education which should be taken into account) (3). Careful evaluation and monitoring for symptoms of VR-induced dizziness is required for safety reasons (3, 21).

On a *mesolevel*, service providers should consider adaptability to enable accessibility, for example, in public transport, and counter the stigmatizing effect of not having access to everyday technologies and consider strategies to enhance participation, e.g., offline and online choices for all public services (BPG, 4). When public services rely on smartphones, tablets, and computers, they should provide alternative non-technological options to avoid excluding some people with dementia (BPG, 4). It is recommended that technologies in public spaces are cognitively enabling, sited in a supportive physical location while continued face-to-face services are available (BPG). Successful implementation hinges on the availability of appropriate equipment and trained personnel (3); promoting videoconferencing to community settings would enhance the accessibility of dementia service to the community (5).

On a *macrolevel*, it is recommended that policymakers are aware of barriers to access, for example, public transport, and to consider adaptations to enable better accessibility (BPG). Policymakers should incentivize the widespread adoption of tablets with pre-installed telehealth software in public healthcare systems (28). Countries may need to make legal provisions to ensure financial services and retailers do not discriminate against people with disabilities regarding payment methods and access to cash (BPG).

#### 3.3.4 Social health domain 2: ability to manage life with some degree of independence

For SH Domain 2, on a *microlevel*, it is also recommended that for the use of “everyday”, “surveillance”, and “cognitive training technology”, individual needs are considered and assistance is provided, based on comprehensive assessment by an occupational therapist (BPG). As online-based cognitive interventions for community dwelling older people with mild cognitive impairment (MCI) or mild dementia are effective in improving cognitive function (i.e., verbal fluency, attention, memory) and psychosocial functioning (i.e., depression, quality of life), it is recommended to provide a multidomain intervention for at least 30 min per session over 6 weeks (5). Monitoring should be used with caution and not without the consent of the person with dementia (11). Exploit information such as Wi-Fi and Bluetooth to detect wandering behavior more accurately (15). Although there is a need for compact Surveillance Technology (11), this does not necessarily mean designers should develop products, which can be covertly used (27). Rather, this recommendation highlights stigmatization that could occur when people with dementia wear visible surveillance technology (27). When technology to support self-management of the person with dementia is implemented, the ecological validity and cultural context in which it will be implemented should be considered, to ensure its applicability in “real-life situations” (BPG). As people with dementia can have difficulty finding apps for self-management, meaningful activities, and social participation that match their needs, interests, and abilities, tools that help them find such apps are recommended (BPG). Nursing staff may consider robotic care for people with dementia due to its clinical benefits (22). Voice and interaction style of socially assistive robots (e.g. Intelligent Cognitive Assistant) should be chosen based on preferences of the user, not those of the caregiver, and the robot should act as an encouraging guide (11).

On a *mesolevel*, it is suggested that nurses can play a pivotal role in improving the quality of life of people with mild dementia by providing or stimulating multidomain digital cognitive interventions (5).

#### 3.3.5 Social health domain 3: participation in social activities

For SH Domain 3, on a *microlevel (and macrolevel)*, it is recommended that individual needs are considered and tailored assistance provided, for example, by facilitators of video meetings and moderators of text-based platforms, such as Facebook groups (BPG). When introducing new technologies, the focus should be on aspects that are likely to encourage people with dementia's interest, such as family photographs, video calls with friends and family, music, games, or art applications (BPG). The incorporation of social interaction elements is also important (BPG). Video calls should be introduced in a way that they are a pleasure to participate in, at an early stage of the disease (11). Tablet-based interventions should be considered as one effective option to support social participation of community-dwelling people with mild dementia, but the choice to provide such an intervention should be based on user characteristics and needs (BPG). Furthermore, a tool that helps people with dementia or caregivers to find suitable apps is recommended, matching interventions to user interest, needs, and ability (BPG). Digital reminiscence therapy is recommended to enable personalized care, reflecting individuals' desires and preferences, thus, contributing to person-centered care (6). It is recommended to create attention for reminiscence group therapy applications among dementia care professionals in care settings, for example through inspiring and informative videos or other online information (6).

On a *mesolevel*, organizations are recommended to continue to utilize video calls and telephones as low-cost, widely accessible communication tools, especially for initial consultations or follow-up care (28). Providing meaningful activities, such as personalized exergaming activities, has proven value for social health, and further implementation, for example in day care facilities, is therefore recommended (BPG). To ensure successful implementation of the technology, multiple employees should be responsible and there must be support from the management of care organizations (BPG). Collaboration between public health organizations and private sector actors should be promoted to facilitate the development and dissemination of cognitive-aerobic training programs, such as Wii Balance Board, in community and rehabilitation centers with the goal of increasing physical health and social engagement (23). Incentive programmes for healthcare professionals should be established to integrate active play into their therapeutic practices, highlighting the social benefits of these activities (23). Implementing music therapy interventions with a more comprehensive assessment of caregivers' profile may be advantageous in supporting people with dementia in long-term care settings (6). Activities that use digital generic photos as the main tool may be easier to set-up in terms of acquiring personal photos and designing social activities around it (24). Robotic platform features and applications need to be tailored to the needs and preferences of end-users before implementing them in community-based dementia care (BPG). Also, when planning to introduce pet robots in long-term care settings, residents with dementia and their family members should be involved (BPG). To minimize potential negative impacts from using pet robots, care providers should assess their suitability for individuals with dementia, and facilitate their use based on each individual's preference, needs, and abilities. As these can fluctuate, care providers should also monitor and re-evaluate the use of pet robots (BPG). Pet robots, like PARO, should not be used to replace staff time, but to comfort people and during periods when staff are occupied (6). Access to the internet should be freely available in care homes so residents with and without dementia gain access to online resources, including social media, entertainment, and information (BPG). It is important that facilitators and moderators of interactive interventions are skilled in communication and listening, and trained to guide technology-based interventions specifically. The choice to provide such an intervention should be based on user characteristics, like severity of dementia, and needs (BPG).

On a *macrolevel*, policymakers are recommended to prioritize the needs of older people with or without dementia to socially participate in the community and provide technological services, preferably with a social interaction element, that address this need to engage in out-of-home activities (8). Policymakers should be motivated to include technology-based health interventions in public initiatives (23).

- *Directions for future research recommended in the 28 reviews, the INDUCT/DISTINCT Best Practice Guidance (BPG) and the INTERDEM position paper* *(**8**)*

The results of the thematic analysis on future research recommendations are presented below. For theme 1, only recommendations were made on a microlevel, for theme 2 on a micro and mesolevel. The recommendations for theme 3, 4, and 5 were all categorized on a mesolevel, and for theme 6 on a macrolevel.

#### 3.3.6 Theme 1: needs-based technology

On a *microlevel*, special attention should be given in prospective research to the development and evaluation of information and communication technology solutions for the most frequently experienced unmet needs: personalized information; compensation for disabilities like memory problems; help needed by people with dementia and their caregivers to cope with the behavioral and psychological changes associated with the dementia; and help needed with daily activities, including pleasant activities (16). Future studies should explore the potential, and evaluate the impact, of technological interventions to promote self-management and reduce social isolation and enhance social participation of community-dwelling older adults with dementia. Studies should also address personal disease-related factors, social and material environmental factors that may facilitate or impede social participation facilitated by technology (8, 19, 25). For example, research should examine the efficacy and safety of smartphones to promote outdoor independence of people with dementia (15), and determine if the effect of virtual reality training on cognition can translate to real-life improvements and better quality of life, considering potential moderators like presence, motivation and engagement and complexity of virtual environments in virtual reality training interventions (21). Moreover, studies need to evaluate the effectiveness of remote consultation, by (low-cost) telephone or video (camera, headphone, tablet), in improving patient engagement and care outcomes. Such studies should consider different healthcare environments, including rural and underserved areas, and explore if remote consultations improve trust and communication in patient-provider relationships, or reduce healthcare disparities in regions with limited specialist availability. Finally, the cost-effectiveness and scalability of remote consultation require attention (28).

#### 3.3.7 Theme 2: user-friendly, personalisable technology, reflecting diversity

On a *microlevel*, future studies on technology should be targeted at the age-related digital divide and health-related conditions, such as sensory impairments that are likely to be experienced by the users (1). Research also needs to ensure that technologies can leverage dementia support and care, and that people with dementia are enabled and empowered to use it (1, 28). ATs should be designed in an intelligent, context-aware, unobtrusive way, which means they should be user-friendly, personalisable, easy to use (14, 16, 17, BPG) and inclusive, i.e. addressing the cognitive usability to reduce the level of challenge, and ensure an optimal user experience with clear signposting and easy and intuitive navigation (BPG). In addition, in case of mobile monitoring technology, they should be wearable, low-cost, and discreet (14). Future research should explore benefits of innovative approaches in delivering tailored tutorials and instructions aligned with cognitive abilities and preferences (18). Developers should also investigate the needs of the people who will be implementing technological interventions after a trial phase, such as case managers, hospital workers, volunteers, or professionals associated with advocacy groups (BPG). For tele-caregiving, user-needs analyses are needed including contextual inquiry, journey maps, decision making models, and workflow assessments (10). Regarding robotic care, interventions frequency, duration, and possible negative outcomes should be investigated (22). Further investigation on pet robots needs to be done into design preferences of people with dementia comparing domestic animals like cats and dogs to designs like a dinosaur and seal (BPG).

On a *mesolevel*, AI-based algorithms that can complement the therapist's proficiency are needed, to compose treatment programs according to the individual patient's cognitive ability (5). The relationship between technology use and the effects on residents of long-term care facilities should be prioritized in research (7). The limited geographical diversity of technology studies, often conducted in USA and UK, hinders generalization and calls for broader global representation to account for cultural and contextual variations (1, 7). Future research should also concentrate on individuality and conflicting needs in different countries. It should be ensured that a new technology is compatible with a range of relevant platforms to promote implementation (BPG). Regarding surveillance technology, designers and other industry and academia stakeholders need to acknowledge user needs (27). Access control technology may be used to increase the opportunity for people with dementia to stay in place in a secure unit, but further exploration is needed of the conditions for use of this technology in the field of dementia care and careful evaluations (25).

#### 3.3.8 Theme 3: methodology (design, development, testing)

Regarding the theme Methodology *(mesolevel)*, sub-themes were identified: i) user involvement, ii) data collection, outcome measures, and research designs, iii) effect evaluations and mechanisms, iv) considering context.

*User involvement*: research and development processes should be user-driven, grounded within a thorough understanding of needs, preferences, and desires of potential users (16). People with dementia or caregivers should be included in studies on the design, development, testing, and implementation of technologies with a diverse user group to make the final product better attuned to their needs, more personalized, and more helpful (0, 1, 10, 11, 16, BPG). Research and experiments in Living Labs should be promoted as they encourage the co-creation of innovative solutions and reliably show the effects of innovative solutions on daily life (9, 16). It may be necessary to develop guidelines on the Living Lab approach, for sustainability of innovative solutions and facilitation of engagement with stakeholders and users (9).

*Data collection, outcome measures, and research designs:* user-relevant outcome measures must be used (0). Established models for user-centered designing should be adapted and validated to be used with people with dementia and guidelines created (20). The process of prototyping and usability testing should be optimized by gathering feedback on working prototypes by means of observation and self-perceived reports of people with dementia (BPG). Moreover, consistent terminology (defining the concept of interest) (8) and standardized methodologies and outcome measures should be used to allow comparisons between interventions and studies (7). Future studies should consider developing and validating an outcome assessment that covers all dimensions of social participation (8, 12). To capture the complex nature of social participation, it would be of value for studies to collect both qualitative and quantitative data (8). A more judicious application is needed of standardized measures and effect sizes that will support clinicians, long-term care managers, and researchers to understand how and which technology can be used to address the immediate and long-term needs of people with dementia and their caregivers at different stages of neurocognitive disorders (1, 7). Use of log data and big data is also recommended to assess usability, effectiveness, and adherence to ATs (20). Finally, the implementation of standards on conducting and reporting research could bring this field of research a great step forward (20).

*Effect evaluations and mechanisms*: more research into the usability, usefulness, and acceptability of technology to promote social health, and larger (randomized) controlled effect trials should be done. Such studies should comprise longer intervention durations and follow-up measurements, than previous studies, and use standardized outcomes, which also consider facilitators and barriers of implementation of the technology (10, 12, 16, 17, 19, 20, 24, 26). Cost-effectiveness research into eHealth interventions should also be considered to inform policymakers to make the right decisions when deploying eHealth interventions (BPG). Regarding exergaming, the creation of more interactive and accessible games, that promote physical activity and social health, should be supported, and research should focus on mechanisms of the games to adapt them to users with various physical abilities (23).More controlled trials into exergaming are needed (23), maintenance of effects (long-term follow-up) need to be investigated, and sensitive and objective measures of daily functioning (including wearable technology; 23) should be used to better understand the significance of cognitive improvements for daily life and effects (of balance exercises) on incidence of falls and fall related injuries (26). Furthermore, mechanisms of impact and interaction effects should be studied (26), while including diverse ethnic groups.

*Considering context:* there is a need to further investigate the technologies available on the market and compare them with the ones found in the literature (27). When evaluating the impact of technology on social health, it is recommended to also evaluate how technology supports people in coping psychologically and emotionally with the consequences of dementia in their daily life, and to consider potential benefits of technologies in family caregivers (BPG). The AT industry should also comply with the evidence standards, and this could promote a more transparent market of evidence-based AT solutions for people with dementia (20).

#### 3.3.9 Theme 4: expertise of researchers

On a *mesolevel*, it is recommended to ensure adequate training for researchers on dementia and inclusive engagement (0). A variety of stakeholders and experts should be involved to ensure that all relevant aspects are met throughout the entire life cycle of AT for people with dementia, from scoping and initial design to dissemination and adoption (20).

#### 3.3.10 Theme 5: ethics

On a *mesolevel*, researchers should apply existing ethical guidelines in their research (0). Monitoring should be used with caution and not without the consent of the person with dementia (11). Designers and healthcare professionals should be aware that stigmatization can occur when people with dementia wear surveillance technology (27).

#### 3.3.11 Theme 6: financing and policy

On a *macrolevel*, resources should be made available for more and larger randomized controlled trials and cost-effectiveness research (0), as this will give a balanced picture of effects and costs and will help policy makers to make the right decisions when deploying technology-based interventions (BPG). It is recommended to make public funding for development and testing conditional on involvement of people with dementia and their caregivers (0). Furthermore, it is suggested that research could focus on policy frameworks for reimbursing telehealth services delivered via tablets and ensuring equitable access, examine the impact of such reimbursements on telehealth adoption and healthcare equity, and evaluate how these tools can reduce healthcare costs while maintaining quality of care (28).

## 4 Conclusions and discussion

By means of an umbrella review we examined 1) how many, and which, technologies are personalisable and effective in one or more of the most frequently mentioned unmet needs related to social health, and 2) what needs to be done, and further investigated, to ensure that the supply of usable and effective technology to promote physical, mental and social health in dementia equitably meets the demand.

### 4.1 Usable and effective personalisable technologies to promote social health

In total there were 329 references to specific technologies in the 28 reviews included. Almost half (48%) were considered to be to some extent personalisable, most commonly by offering adjustable settings, for example, in how screen-based applications were displayed, or in flexible content, such as, the option to add or remove specific apps or features to a software-based technology. Personalization was reported to be necessary, and in some cases was provided, in response to variations on factors related to the person and their life history. As such, this personalization referred to adapting the technology to match the individual's own life history, culture, age, interests, religion, level of digital literacy, and physical and mental health status. The latter included tailoring to the severity of cognitive impairment, presence of dementia-related symptoms such as apathy or agitation, and/or physical fitness. However, more than half of references technologies did not allow for any such personalization.

In relation to the three domains of social health, more than half (58%) of the referenced technologies that could support the “Capacity to fulfill one's potential and obligations” were considered personalisable, and half of the referenced technologies that could support “Managing life with some degree of independence” (50%) or “Participation in social and other meaningful activities” (48%).

Of a small majority (59%) of the referenced technologies, information about their usability was found, though generally only limited details were provided in the reviews and original articles. In a minority (23%) of the referenced personalisable technologies, evidence for effectiveness from at least one RCT was reported. In most cases (*n* = 15, 56%), this concerned technologies relevant to managing life with some degree of independence, while somewhat less concerned technologies relevant to supporting the capacity to fulfill one's potential and obligations (*n* = 11, 41%) or supporting participation in social and other meaningful activities (*n* = 9, 33%). Of the in total 36 conducted RCTs, 27 (75%) showed evidence for effectiveness.

**Figure 2:**
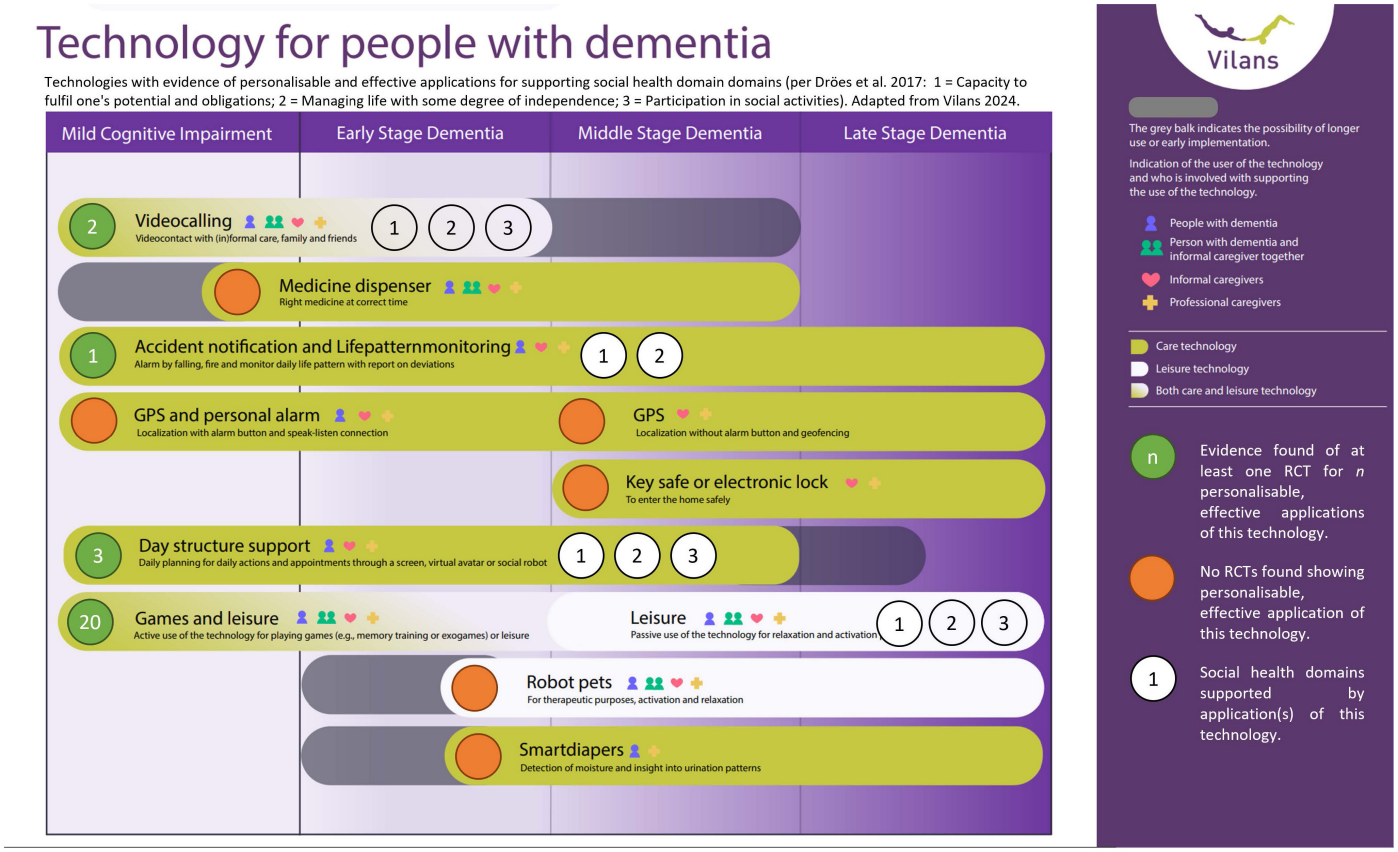
Technologies with evidence of personalisable and effective applications for supporting social health domains (per 2: 1 = Capacity to fulfill one's potential and obligations; 2 = Managing life with some degree of independence; 3 = Participation in social activities). Adapted from 13. Note: 26 of the 27 technologies with RCT evidence were mapped to the Vilans model. One intervention (WESIHAT 2.0), a purely health information based intervention, did not map to any of the categories in this model.

In Figure 2, we summarized the findings of our research, integrated into an infographic of an ecosystem of personalisable, usable, effective, and implementable technologies for supporting social health in the different stages of dementia. The infographic on available different types of technologies for different stages in dementia was originally developed by Vilans, a knowledge organization for care and support in the Netherlands (13). The green bullets with numbers indicate that evidence was found for effectiveness in one or more RCT into personalisable technologies in a category, the orange bullet indicate that no RCT were found. The white bullets refer to the corresponding social health domains supported by the technologies in a category.

### 4.2 Discussion of the findings through the lens of equitably meeting the demands of people with dementia

The findings of our review show that almost half of the technologies that can promote social health are personalisable to some degree, and of those a small majority seems usable for people with dementia. However, for only a minority of the referenced personalisable technologies is there evidence for effectiveness associated with one or more of the social health domains, as evaluated in an RCT. This is in line with the findings of the systematic review of 8, although there has been an increase in RCTs since then, as evidenced by the fact that the majority of RCTs were conducted after 2017. Nevertheless, many studies used one group designs and small samples, which makes it difficult to draw firm conclusions about the effectiveness of the evaluated technologies (see also (24)). At the same time, while RCTs provide valuable insights into the effectiveness of technological interventions, the limitations of RCTs should be kept in mind (34), also allowing for various types and sources of evidence to impact practice and policy (35). Given the numerous and varied recommendations provided for best practice and effective implementation of technologies, often implicitly indicating the gaps that still need to be fulfilled, it is clear that this field is complex, that the practice is still far from meeting the set requirements for successful implementation. There is a lot that needs to be done to ensure that the supply of usable and effective technology to promote physical, mental, and social functioning relevant to social health in dementia equitably meets the demand.

In this regard, on a *microlevel*, technological developers and care providers are recommended to individualize implementation, test ecological validity, and personalize technologies, in line with user characteristics including age, interest, religion, digital literacy, physical, and mental health status, as well as user needs, preferences, abilities and diversity in people and contexts. Only in this way can technology contribute to fulfilling unmet needs regarding social health (see also (32)). Urgent attention is asked for the prevention of discrimination by ensuring that public technological systems are usable for customers living with dementia. Furthermore, the use of tools that help people with dementia find apps for self-management, meaningful activities, and social participation that match their needs, interest, and abilities are recommended. On a *mesolevel*, service providers are strongly recommended to consider adaptability to enable accessibility and to provide alternative offline options to avoid excluding people with dementia. Also, the need of informed and trained personnel, (continuous) clinical evaluation of the usefulness, acceptability, and effectiveness of technological devices is recommended. The acquired knowledge on this process should be included in professional guidelines to ensure that people with dementia and carers receive up-to-date, evidence based, inclusive quality care, and support (36). Updating guidelines may help the further uptake of technologies available for people with dementia and carers as a review showed that there is very little evidence of widespread practical application of such technologies. Instead, people with dementia and carers frequently still rely on everyday technologies re-purposed to meet their needs (37). Finally, on a *macrolevel*, policymakers are recommended to prioritize addressing the need of older adults to participate in the community and provide technological and other services to address this. Policymakers need to be aware of barriers to access of public services like public transport and financial services, consider adaptations to enable better accessibility, and establish national standards and guidelines for, for example, communication technology. Countries may need to make legal provisions to ensure financial services and retailers do not discriminate against people with disabilities regarding payment methods and access to cash.

### 4.3 Strengths and limitations of the study

To our knowledge, this is the first study on the personalisability of ATs used in dementia care that can promote social health of people with dementia, the usability of personalisable technologies, their effectiveness, and what needs to be done to further implement and develop effective and usable personalisable technologies. Although we originally intended to only review published reviews for this study, we additionally screened original articles as the reviews did not always clearly indicate whether and how the technologies were personalisable. In most cases, we succeeded in accessing the abstracts and/or the full original articles to collect this information.

Besides these strengths, this study also had some limitations that need to be mentioned. First, as an umbrella review with a defined scope, we restricted the number of reviews by including only those explicitly addressing assistive technologies in relation to social health in dementia. Nevertheless, by including recent reviews specifically focused on technology for social health from the INDUCT and DISTINCT Networks as well as both earlier and more recent reviewsidentified through the systematic reviews conducted by the INTEREST Needs Subgroup and the INTERDEM Technology Taskforce, we are confident that our findings broadly reflect the state-of-the-art of personalisable technologies aimed at promoting social health in dementia.

A second limitation is that we limited our review to personalisable technologies that investigated outcomes in one or more of the three domains of social health. This restriction means that no detailed data on usability and effectiveness was collected and analyzed of studied technologies that purely focused on factors that could influence social health, like digital training of cognitive functions, without evaluating their effect on outcomes associated with one or more of the three social health domains. Further research is needed to understand if and how such technologies benefit social health.

A third limitation is that because of our main focus on people with dementia, we excluded technologies that exclusively focused on informal or professional caregivers or care systems, such as online carer support groups, digital education of professional caregivers or mobile accessibility of electronic patient files. Nonetheless, such technologies may be indirectly beneficial for the social health of people with dementia.

A final limitation is that we included only reviews that were published in the English language, what may have influenced our findings. It is possible that relevant recommendations on the subject of study, provided in reviews published in other languages, may have been missed.

### 4.4 Recommendations for practice and research based on this review

In the reviews included and the INDUCT/DISTINCT Best Practice Guidance for Human Interaction with Technologies in Dementia, recommendations were provided on how to promote successful implementation and adoption of effective personalisable and usable technology on a micro-, meso-, and macrolevel. Directions for future research focused on the further development of needs-based technologies that are user-friendly, personalisable, and reflecting diversity, improved research methodologies, expertise of researchers, ethics, and funding and policy. Looking through the lens of equity, to see what can be done to ensure that the supply of usable and effective technology promoting physical, mental, and/or social health in dementia equitably meets the demand, overall, the following can be recommended for implementation in practice and future research (see Box 1):

Box 1 Recommendations for implementation of technologies and directions for future research.

**Regarding implementation in practice:**

On a *microlevel*,

• Implementation of technologies should be individualized, technologies should be ecologically validated and personalisable, in line with user characteristics (e.g. age, interest, religion, digital literacy, physical and mental health status), user needs, preferences, abilities and diversity in people and contexts.

• Discrimination should be prevented by ensuring that public technological systems are usable for customers living with dementia.

• Tools that help people with dementia find apps for self-management, meaningful activities and social participation that match their needs, interest and abilities are recommended

On a *mesolevel*,

• Service providers are strongly recommended to consider adaptability to enable accessibility and to provide alternative non-ICT options to avoid excluding people with dementia.

• Professionals using technologies should be informed and trained, and (continuously) evaluate the usefulness, acceptability and effectiveness of technological devices for the individual with dementia. Scientific knowledge on this should be included in professional guidelines to ensure that people with dementia and carers receive evidence based, inclusive quality care and support

On a *macrolevel*,

• Policymakers should prioritize the need of older adults to participate in the community and provide technological and other services to address this.

• Policymakers should be (made) aware of barriers to access of public services, consider adaptations to enable better accessibility and establish national standards and guidelines for, for example, communication technology, while countries may need to make legal provisions to ensure that services do not discriminate against people with disabilities.

Regarding future research: On a *microlevel*,

• Technologies should be developed, that not only addresses the most frequently experienced unmet needs of people with dementia, but are also suitable, i.e. user-friendly and personalisable, for diverse groups in relation to age, ability, preferences, cultural background and context.

• Researchers should adopt and clearly report robust methods for evaluating usability of ATs, grounded in appropriate theoretical frameworks from human factors engineering and human computer interaction.

• Research should explore if and how personal, disease-related, social and material environmental factors facilitate or impede social health.

• The needs of people who will be implementing the technological interventions in practice should be investigated as they could contribute to equitably meeting the demand.

On a *mesolevel*,

• Research should develop and adopt improved research methodologies (user-driven, user-centred, Living Lab) and guidelines for this.

• More research should be done with diverse user groups and broader global representation to account for cultural and contextual variations, into the usability, usefulness, acceptability and effectiveness of technology to promote social health, Research into mechanisms of impact and interaction effects should be done, as well as into facilitators and barriers of implementation of the technology.

• Evaluation research should use standardized measures (and provide effect sizes) to enable a more judicious application of outcomes by clinicians, LTC managers and researchers when trying to understand how and which technology can be used to overcome the immediate and long-term needs of people with dementia and their supporters at different stages of the disease.

• A variety of stakeholders and experts should be involved in technology research to ensure that all relevant aspects are met throughout the entire life cycle of AT for people with dementia, from scoping and initial design to dissemination and adoption.

On a *macrolevel*,

• Make public funding for development and testing conditional on involvement of people with dementia and their supporters.

• Investigate policy frameworks for reimbursing telehealth services delivered via tablets and ensuring equitable access, examine the impact of such reimbursements on telehealth adoption and healthcare equity, and evaluate how these tools can reduce healthcare costs while maintaining quality of care.
